# Implications of Vascular Depression for Successful Cognitive Aging in HIV disease

**DOI:** 10.21203/rs.3.rs-3154022/v1

**Published:** 2023-07-31

**Authors:** Andrea I Mustafa, Ilex Beltran-Najera, Darrian Evans, Alexandria Bartlett, Vonetta M Dotson, Steven Paul Woods

**Affiliations:** University of Houston; University of Houston; University of Houston; Georgia State University; Georgia State University; University of Houston

**Keywords:** infectious disease, mood disorder, positive psychology, cardiovascular, geropsychology, neurocognitive disorder

## Abstract

**Introduction:**

Although older adults with HIV are at high risk for mild neurocognitive disorders, a subset experience successful cognitive aging (SCA). HIV is associated with an increased risk of vascular depression (VasDep), which can affect cognitive and daily functioning. The current study examined whether VasDep impedes SCA among older adults with HIV.

**Methods:**

136 persons with HIV aged 50 years and older were classified as either SCA+ (n=37) or SCA− (n=99) based on a battery of demographically adjusted neurocognitive tests and self-reported cognitive symptoms. Participants were also stratified on the presence of vascular disease (e.g., hypertension) and current depression as determined by the Composite International Diagnostic Interview and the Depression/Dejection scale of the Profile of Mood States.

**Results:**

A Cochran-Armitage test revealed a significant additive effect of vascular disease and depression on SCA in this sample of older adults with HIV (*z*=4.13, *p*<.0001). Individuals with VasDep had the lowest frequency of SCA+ (0%), which differed significantly from the group with only vascular disease (30%, OR=0.04, CI=0.002,0.68)) and the group with neither vascular disease nor depression (47% OR =0.02, CI=0.33,0.001). Findings were not confounded by demographics, HIV disease severity, or other psychiatric and medical factors (*ps*>.05).

**Discussion:**

These data suggest that presence of VasDep may be a barrier to SCA in older adults with HIV disease. Prospective, longitudinal studies with neuroimaging-based operationalizations of VasDep are needed to further clarify this risk factor’s role in the maintenance of cognitive and brain health in persons with HIV disease.

## Introduction

It is currently estimated that more than half of people infected with HIV in the United States are over the age of 50 years (CDC 2022). The prevalence of older persons living with HIV (PLWH) is likely to continue to rise in coming years, as younger adults with HIV who can access and adhere to modern antiretroviral therapies are living longer and older adults represent approximately 10% of new HIV diagnoses (CDC 2022). In general, older PLWH are at greater risk of immune senescence, medical comorbidities (e.g., vascular disease), and polypharmacy (e.g., Langebeek et al. 2017; Rodriguez-Penney et al. 2013). Older PLWH are therefore more susceptible to frailty (Guaraldi et al. 2011; Bloch 2018), poorer daily functioning (Oursler et al. 2011), lower quality of life (Basavaraj et al. 2010), and mortality (CDC 2022). Yet there is considerable heterogeneity in the clinical course of HIV disease among older adults. A meaningful subset of older PLWH experience successful aging in one or more domains, such as biological health, cognitive efficiency, mental health, social competence, productivity, personal control, and life satisfaction (Vance et al. 2019). As such, it is important to understand the facilitators and barriers for different aspects of successful aging for older PLWH.

The current study focuses specifically on successful cognitive aging (SCA) among older PLWH. The central nervous system (CNS) is vulnerable to the combined effects of HIV and aging. There is evidence of both accelerated and accentuated brain aging in the setting of HIV disease (Peterson et al. 2021; Sheppard et al. 2017). Neuroimaging studies show evidence of the combined effects of HIV and aging on white matter integrity (Kuhn et al. 2018) and cortical and subcortical volume loss (e.g., Guha et al. 2016). Older PLWH are at greater risk for neurocognitive deficits (Deng et al. 2021), particularly in the domains of declarative memory (Woods et al. 2010), executive functions (e.g., Iudicello et al. 2012; Müller-Oehring et al. 2022), and motor abilities (e.g., DeVaughn et al. 2015; Tierney et al. 2019). Moreover, the frequency (e.g., Valcour et al. 2004) and incidence (e.g., Sheppard et al. 2015a) of prodromal (e.g., mild cognitive impairment; Sheppard et al. 2015b; 2019) and syndromic neurocognitive disorders tends to be higher in older PLWH.

Despite the additive effects of age and HIV on the CNS, there are still considerable differences in cognitive outcomes among older PLWH. Indeed, not all older PLWH will develop cognitive deficits, and many will age quite “successfully” in this regard (Malaspina et al. 2011; Saloner et al. 2019). Malaspina and colleagues (2011) were the first to describe SCA among PLWH, reporting that approximately one-third of PLWH were without cognitive symptoms in daily life and showed normatively intact performance on a cognitive battery. Subsequent literature suggests that the frequency of SCA is lower in PLWH as compared to seronegative older adults, with an estimated prevalence of between 19% (Moore et al., 2014) and 32% (Malaspina et al., 2011). Not surprisingly, SCA is associated with better everyday functioning, including medication adherence and healthcare self-efficacy (e.g., Malaspina et al. 2011). Other correlates of SCA include better emotional well-being (Moore et al. 2014), resilience (Mayo et al. 2022; Rubtsova et al. 2019), and optimism (Rubtsova et al. 2019).

Depression and vascular disease may play an important role in SCA in PLWH. Depression is one of the most prevalent neuropsychiatric disorders among PLWH (Chichetto et al. 2021; Dew et al. 1997; Rabkin 2008) and has been associated with greater disease progression and mortality (So-Armah et al. 2020). Similarly, vascular disease is a leading cause of morbidity and mortality in HIV (Miller et al. 2014), and by the year 2030, it is estimated that almost 80% of older individuals with HIV will have vascular disease (Smit et al. 2015). PLWH with depression show greater incidence of carotid plaques (Levy et al. 2020; Parruti et al. 2013), hypertension (Castilho et al. 2020), and stroke (Sico et al. 2021). Furthermore, biomarkers of vascular disease (e.g., glucose, hemoglobin A1C) have been associated with depression in PLWH. Both depression (Bryant et al. 2015; Shimizu et al. 2011) and vascular disease (Cysique and Brew 2019; McCutchan et al. 2012) have been linked to neurocognitive impairment in PLWH, but little is known about their combined influence on the structure and function of the CNS, including SCA.

The co-occurrence of vascular disease and depression in older adults led Alexopoulos and colleagues (1997) to propose the vascular depression hypothesis (VasDep), which suggests that vascular disease may “predispose, precipitate, or perpetuate” geriatric depression. The VasDep hypothesis specifically proposes that some aspects of depression result from disrupted mood-related brain networks (i.e., frontal-subcortical circuits) by way of white matter lesions (Krishnan et al. 1997). It is also plausible that depression may increase risk of vascular disease and/or exacerbate pre-existing vascular disease-related conditions that then further amplify depressive symptoms (Kirton et al. 2014; Dotson et al. 2013). Of clinical relevance, individuals with VasDep have greater risk of functional disability and cognitive impairment (e.g., executive dysfunction and slowed processing speed) relative to individuals with non-vascular depression (Aizenstein et al. 2016). It is estimated that 3.4% of adults met criteria for VasDep (González et al. 2012), with the frequency being higher among middle-aged and older persons with a lifetime history of major depressive disorder (MDD) and vascular disease comorbidity. Additionally, persons who met criteria for VasDep demonstrated increased disease burden relative to non-depression and major depression alone populations.

VasDep may be common and impactful among PLWH. In a cross-sectional study, Beltran-Najera and colleagues (2023) reported on the frequency and everyday functioning implications of VasDep in 536 PLWH and 272 seronegative individuals. VasDep was operationalized as the presence of two or more vascular conditions and depression, as determined by a normative elevation on the Depression/Dejection subscale of the Profile of Mood States (POMS; McNair et al. 1981) or a diagnosis of MDD per the Composite International Diagnostic Interview (CIDI; World Health Organization 1998). Findings indicated that PLWH had a three-fold increased rate of VasDep (15.7%) relative to seronegative individuals (4.8%). Among PLWH, VasDep was associated with worse historical HIV disease markers (e.g., lower nadir CD4 counts) and older age; furthermore, PLWH with VasDep were over 5 times more likely to be dependent in everyday functioning as compared to PLWH with either vascular disease or depression alone.

As such, it is plausible that VasDep may play a role in SCA among older PLWH. Further support for the contributions of both vascular disease and depression in SCA is evident in studies in healthy adults and PLWH. For example, lifestyle factors that are known to increase risk of vascular disease (e.g., poor diet, lack of exercise, and smoking) are associated with a reduced likelihood of SCA in seronegative samples (Aguero-Torres et al. 2001; Tomaszewski Farias et al. 2009). The scant literature on vascular disease, depression, and SCA among PLWH is mixed thus far. Some studies show a positive association between SCA and vascular disease (Moore et al. 2018; Wallace et al. 2017) and depression (e.g., Malaspina et al. 2011; Moore et al. 2018), whereas others report null findings (e.g., Moore et al. 2014). However, no studies have examined these two clinical variables in combination through the lens of VasDep in relationship to SCA. In the present study, we aimed to investigate the effects of VasDep on SCA in older adults with HIV. Considering the above-reviewed literature and findings from our prior study (Beltran-Najera et al. 2023), we hypothesized that older PLWH with VasDep would demonstrate lower rates of SCA as compared to older PLWH without VasDep.

## Methods

### Participants

The study sample included 140 PLWH aged 50 and older who were enrolled in a study on prospective memory (Woods et al. 2020). Aspects of these data have been published (e.g., Thompson et al. 2022); however, the analyses of VasDep are novel. Individuals were recruited via community-based organizations, advertisements, and local clinics and enrolled through the University of California San Diego’s (USCD) HIV Neurobehavioral Research Program. Participants were excluded from the study if there was a positive history of serious psychiatric disorders (e.g., schizophrenia), color blindness, intellectual disability, neurological conditions not due to HIV disease (e.g., non-HIV-related dementia, seizure disorders), head injury with loss of consciousness greater than 30 minutes, current substance dependence as measured by the CIDI version 2.128 (World Health Organization 1998), or positive urine toxicology screen/breathalyzer for illicit drugs on the day of testing. All participants provided written, informed consent prior to completing a comprehensive medical, psychiatric, and neuropsychological assessment that was approved by the institutional review board.

### Materials and Procedure

#### Operationalizing Sucessful Cognitive Aging

Successful cognitive aging (SCA+) in HIV is often defined as the absence of cognitive impairment and/or cognitive symptoms in daily life (Malaspina et al. 2011; Moore et al. 2014; Wallace et al. 2017; Moore et al. 2018; Fazeli et al. 2020). This definition was applied in the present study to demarcate PLWH with SCA+ (n = 37) and SCA− comparison participants (*n* = 99). Specifically, participants scoring within normal limits on a full neurocognitive battery (Casaletto et al. 2015; www.cogstate.com) and scores < 1.5 standard deviations (SD) above the normative mean on the Frontal Systems Behavior Scale (FrSBe; Grace & Malloy 2001) disinhibition and executive function subscales, POMS (McNair et al. 1981) Confusion/Bewilderment subscale, and the Prospective and Retrospective Memory Questionnaire (PRMQ; Smith et al. 2000) were classified as SCA+ (*n* = 37). The remaining 99 individuals with neurocognitive impairment (*n* = 44) and/or elevated cognitive symptoms in daily life (*n* = 55) were assigned to the SCA− comparison group.

#### Operationalizing Vascular Disease, Depression, and Vascular Depression

##### Vascular Disease.

The presence of current and/or historical vascular conditions was determined in an interview conducted by the research nurse. Specifically, participants were asked to identify any history of chronic pulmonary disease, congestive heart failure, transient ischemic attack, type 2 diabetes mellitus, hyperlipidemia, hypertension, myocardial infarction, and peripheral vascular disease. Participants received a score of 1 for each historical vascular condition endorsed (sample range = 0–9) and a score of 2 for a current vascular condition (sample range = 0–4). Participants with a total weighted score of 2 or more (range = 0–9) were assigned to the vascular group (*n* = 104), which is consistent with prior research (Bogoian & Dotson 2022).

##### Depression.

Depression was operationalized using the CIDI (World Health Organization 1998) and the 15-item Depression/Dejection scale of the POMS (McNair et al. 1981), which are two well-validated measures of depression (Patterson et al. 2006). The CIDI is a structured lay interview for MDD. On the POMS, participants indicated how they have been feeling over the past week (e.g., unhappy, sorry for things done) using a five-point Likert-type scale ranging from zero (“not at all”) to four (“extremely”). Possible scores ranged from 0 to 60. POMS scores were transformed into z-scores using age- and sex-based normative data (Nyenhuis et al. 1999), such that higher z-scores reflected worse depressive symptoms. Individuals who obtained a score of 1.5 or higher on the POMS Depression/Dejection subscale or met criteria for current MDD based on the CIDI were classified as depressed (Patterson et al. 2006). We used two indicators of depression because the frequency of current MDD diagnoses per the CIDI was fairly low and the multi-modal assessment approach bolsters the rigor of the measurement.

##### Vascular Depression.

Participants were stratified based on vascular factors and depression status, which produced sufficient samples of persons: (1) without vascular disease or depression (V−D−) (*n* = 32), (2) with vascular disease, but without depression (V+D−) (*n* = 74), and (3) with both vascular disease and depression (V+D+) (*n* = 30). Three participants did not meet criteria for vascular disease but met criteria for depression (V−D+), however due to such a small sample size this group was excluded from the present study. One participant was missing vascular and depression data and was excluded.

#### Statistical Analyses

All statistical analyses for the present study were performed in JMP Pro version 16.0 (SAS Institute Inc.) with a critical alpha of .05. Covariates were determined using a data-driven approach (Field-Fote 2019) in which any sociodemographic or clinical variable (see Table 1) that significantly related to both the independent and dependent variables, as determined by a series of t-tests or chi-square analyses, were included as a covariate. The Cochran-Armitage test for trends (Armitage 1955) was used to determine if depression and vascular health risks conferred additive detrimental effects on SCA. Follow-up chi-square analyses were conducted to identify significant differences in frequencies of VasDep among those who are SCA+ and SCA−. Finally, a series of logistic regressions were conducted to investigate whether SCA status predicted POMS depression scores and/or number of vascular-related health conditions.

## Results

**Figure 1:**
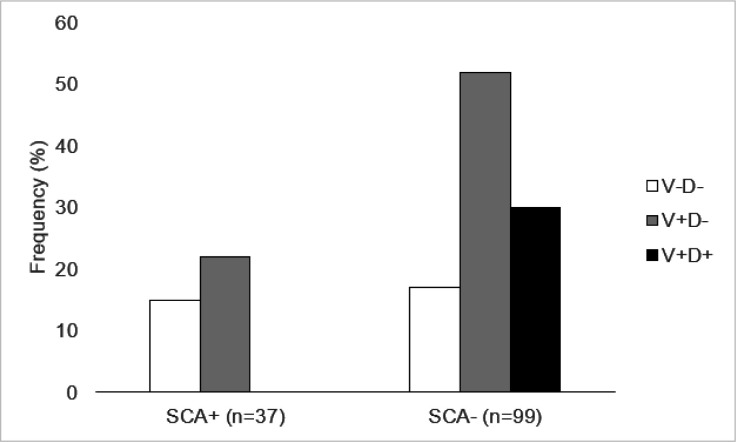
Individuals in the V+D+ group were less likely to be in the SCA+ group compared to the V+D− and V−D− groups. Three participants did not meet criteria for vascular disease but met criteria for depression (V−D+), however due to such a small sample size this group was excluded from the present study. Note: V+D+ = vascular health risks and depression; V+D− = vascular health risks, no depression; V−D− = no vascular health risk factors, no depression; SCA+ = successful cognitive aging; SCA− = not successful cognitive aging

No significant differences were identified among participants in the V−D−, V + D−, or V + D + groups on any sociodemographic or clinical variables listed in Table 1 (see Table 2; *ps* > .05); therefore, no covariates were necessary in the primary analyses. A Cochran-Armitage test for trends revealed a significant additive trend of VasDep group and SCA+/− status (*z* = 4.13, *p* < .0001). Specifically, individuals with vascular disease and depression (V + D+) had the lowest rates of SCA+ (Fig. 1), which significantly differed from individuals with only vascular disease [(V + D−); (*χ*^2^ = 17.26, *p* < .0001, odds ratio (OR) = 0.04, confidence interval (CI) = 0.002,0.68)] and individuals with neither vascular disease nor depression [(V−D−); *χ*^2^= 17.70, *p* = .0001; OR = 0.02, CI = 0.33, 0.001)]. While fewer V + D− participants were in the SCA+ group relative to V−D− participants (*n* = 15, *n* = 22, respectively), these groups did not significantly differ in SCA+/− grouping (*χ*^2^ = 2.83, *p* = 0.092; OR = 0.50, CI = 0.20, 1.23).

A logistic regression was conducted to examine the independent effects of a number of vascular health risks and POMS Depression-Dejection subscale scores on SCA+/− groups. The overall regression model was significant (*χ*^2^(2, N = 136) = 21.60, *p* < .0001) and both variables were independent, significant contributors. POMS Depression-Dejection subscale scores were a significant predictor of SCA+/− group (*χ*^2^ = 32.60, *p* < .0001; OR = 0.82, CI = 0.74–0.91). Therefore, for every decrease in POMS Depression-Dejection subscale scores, individuals had a 1.2 increase in odds of being in the SCA + group. Similarly, number of vascular health risks significantly predicted SCA+/− groups (*χ*^2^ = 5.69, *p* = .017; OR = 0.80, CI = 0.66–0.97). Furthermore, for every decrease in endorsed vascular health risks, individuals had a 1.3 increase in odds of being in the SCA + group.

## Discussion

Given the rising prevalence of older PLWH with the success of modern antiretroviral therapies, it is imperative to understand the modifiers of SCA in the context of HIV disease. While depression and vascular conditions are common among PLWH and are each associated with increased risk of morbidity and mortality (Miller et al. 2014; Sheppard et al. 2017; So-Armah et al. 2020), little is known about their individual and combined contributions to SCA in PLWH. The present study is the first to find evidence of an additive effect of vascular disease and depression on SCA among PLWH. Older adults with HIV with comorbid vascular disease and depression demonstrated the lowest rates of SCA compared to those with HIV and vascular disease alone and to individuals with neither vascular disease nor depression. This novel finding highlights potential intervention targets to promote healthy cognitive aging in PLWH.

Findings from this study extend prior work showing that vascular disease and depression are associated with cognitive deficits in PLWH (Bryant et al. 2015; Cysique & Brew 2019; McCutchan et al. 2012; Shimizu et al. 2011). Consistent with the literature, the present study found that having fewer depressive symptoms and fewer vascular risk factors increased the odds of SCA. This complements previous findings by focusing on the association between the absence of risk factors and positive outcomes rather than the presence of risk factors and negative outcomes. Absence of depression has been previously associated with self-rated successful aging in PLWH aged 56 to 65 (Rooney et al. 2019); however, participants in that study self-reported successful aging on a one-item measure based on their own conceptualization. Our outcome of SCA captures optimal aging in a more comprehensive manner than self-report of subjective successful aging or examination of single cognitive tests or domains, as the operational definition encompassed intact performance across cognitive domains as well as scores < 1.5 SD above the mean on self-reported executive dysfunction, memory slips in everyday life, and mood.

In addition to the independent relationships of depression and vascular disease with SCA, we found that individuals with VasDep, based on comorbid depression and vascular disease, were least likely to be in the SCA + group. This was true in comparison to individuals without depression or vascular risk as well as those with vascular disease but no depression. The latter suggests that depression confers a risk to cognitive health in PLWH beyond vascular disease alone, mirroring our previous finding that VasDep in PLWH increases the risk for functional disability to a greater degree than depression or vascular disease alone (Beltran-Najera et al. 2023). These results are also consistent with the broader VasDep literature showing greater cognitive deficits, functional disability, and brain changes in older adults with VasDep compared to older adults with depression in the absence of significant vascular risk (Jellinger 2021).

VasDep is thought to result from the disruption of fronto-subcortical circuits by white matter lesions caused by chronic vascular conditions (Alexopoulos et al. 1997; Krishnan et al. 1997). These neural networks connect regions of the frontal cortex with deeper subcortical structures to modulate cognitive and affective processes such as executive functions, inhibitory control, motivation, and motor movements (Bonelli & Cummings 2007). Damage to any one of these networks could therefore result in mood symptoms as well as a range of impairments that could have significant impacts on daily functioning, which may explain the disproportionate cognitive and functional disability often seen with VasDep (Aizenstein et al. 2016; Alexopoulos et al. 1997). A key subcortical structure within these loops is the basal ganglia which, in addition to having a primary role in motor functioning, is also a key region in the limbic system and thus plays a role in mood and emotion regulation. It stands to reason that disturbances along basal ganglia-related pathways may be involved in the development of VasDep. This is of particular relevance within the context of HIV disease since the virus is known to disproportionately impact dopamine-rich structures such as the basal ganglia (DeVaughn et al. 2015).

Moreover, even among PLWH with well controlled viral loads using antiretroviral treatments, the risk of developing vascular diseases (and consequently VasDep) remains elevated compared with seronegative individuals due to chronic immune activation and inflammation (McIntosh et al. 2021) as well as the vascular side effects from the medications themselves (DeVaughn et al. 2015). Inflammation is a core characteristic of HIV (Deeks et al. 2013) that negatively impacts several aspects of health, including gut permeability, renal function, and vascular function (Duprez et al. 2012; Neuhaus et al. 2010; Siedner et al. 2018). Elevated pro-inflammatory cytokines are also associated with depressive symptoms in older adults with HIV (Derry et al. 2022). HIV exacerbates inflammaging, systemic inflammation due to aging reflected in high levels of pro-inflammatory cytokines that also contributes to multiple chronic disease, such as type 2 diabetes, sarcopenia, dementia, vascular disease, and depression (Babu et al. 2019; Ferrucci & Fabbri 2018). Inflammation not only contributes to vascular disease, which in turn can lead to cognitive impairment, but inflammatory processes have also been directly linked to cognitive deficits in a range of populations, including PLWH (Rubin et al. 2018).

The interrelationships between vascular disease, depression, inflammation, and cognitive functioning suggest SCA may be promoted in PLWH by prevention and treatment efforts that reduce inflammation and vascular risk. Physical activity and diet are particularly important. Higher levels of physical activity are associated with benefits to brain health, general health, and mental health across the lifespan, including in older adults and in PLWH (Erlandson et al. 2021; Fazeli et al. 2023; Ibeneme et al. 2022). Physical activity also reduces chronic inflammation (Monteiro-Junior et al. 2018), reduces white matter lesions in the brain, and leads to other direct benefits to the brain such as increasing regional volumes and promoting the production of brain-derived neurotrophic factors (Umegaki et al. 2021). There is evidence that PLWH have lower levels of physical activity, and that physical inactivity is associated with higher levels of depressive symptoms (Qin et al. 2022). In the same study, depressive symptoms and physical activity mediated the association between HIV status and cognitive functioning. Thus, promoting physical activity may promote SCA in VasDep by reducing risk factors for vascular disease and depression and by directly benefiting brain health.

Similarly, diet and nutrition may support vascular health and reduce inflammation. A diet rich in antioxidants has been recommended for cognitive health in PLWH due to the role of antioxidants in reducing systemic neuroinflammation (Vance et al. 2022). Heart-healthy diets such as Mediterranean-style diets are associated with better vascular health and brain health in older adults (Roman et al. 2019) and could be promoted in PLWH as well. In support of the potential benefits of heart-healthy diets in PLWH, a randomized parallel-group pilot trial found that compared to general dietary advice to reduce saturated fat intake, PLWH who received support to adopt a Mediterranean-style diet combined with cholesterol-lowering foods for six months had lower LDL-cholesterol and systolic blood pressure after the intervention (Stradling et al. 2021). More research is needed in this limited area to determine the long-term benefits of different dietary patterns on the mechanisms that contribute to vascular disease and depression in PLWH.

PLWH are one of several groups of individuals who face barriers to SCA, including physiological effects of chronic diseases and treatments, a higher burden of chronic stress, and obstacles such as stigma that result in healthcare disparities. These factors can be compounded in older individuals with HIV who not only have a lifetime of accumulated risk factors but also face additional barriers resulting from ageism, as well as biological risks related to aging. The lack of data related to these important considerations is a limitation of the current study, as is the predominance of male, White, highly educated participants in our sample. Longitudinal studies in demographically diverse samples will further clarify the impact of VasDep on SCA in diverse PLWH and the potential for different behavioral and somatic interventions to improve cognitive outcomes. There is clearly no simple solution that can guarantee SCA in older PLWH; however, our finding that vascular disease and depression independently and in combination impact SCA suggest vascular and depression risk reduction are key targets for treatment. Results also suggest a particular need for clinicians to assess for vascular disease or depression in PLWH who present with one or the other condition, since the presence of both is associated with a lower likelihood of SCA.

## Figures and Tables

**Table 1.** Demographic and clinical characteristics of 136 PLWH with and without vascular disease and depression^a^

	V−D−	V+D−	V+D+	*p*
N = 136	32	74	30	
*Demographics*				
Sex (% women)	15.6	13.5	13.3	0.954
Race/Ethnicity (%)				0.535
Black	25.0	23.0	16.7	
Hispanic/Latin@	9.4	18.9	16.7	
White	65.6	55.4	66.7	
Other	0.0	2.7	0.0	
Age (years)	54.5 (5.2)	58.1 (6.1)	57.5 (6.8)	0.025
Education (years)	13.9 (2.9)	14.0 (2.6)	14.3 (2.6)	0.837
*Psychiatric*				
Generalized Anxiety Disorder (%)	6.3	16.2	23.3	0.144
Substance Use Disorder (%)	71.9	66.2	83.3	0.195
*Medical*				
Non-vascular medical conditions	0.4 (0.6)	0.5 (0.7)	0.5 (0.8)	0.869
Estimated HIV duration (years)	22.2 (8.9)	20.7 (8.4)	23.4 (7.2)	0.315
Plasma HIV RNA (% detectable)	9.7	1.5	3.5	0.179
Current CD4 count (cells/μL)^2^	581.8 (349.2)	688.0 (279.1)	696.0 (304.4)	0.211
Nadir CD4 count (cells/μL) ^2^	169.3 (159.6)	190.2 (168.4)	186.1 (206.4)	0.852
AIDS (%)	59.4	67.6	82.8	0.111
On ART (%)	90.6	94.4	93.1	0.783

a Values are means (standard deviation) or valid sample % values. PLWH = people living with Human Immunodeficiency Virus; V = vascular; D = depression; RNA = Ribonucleic acid; CD4 = Cluster of differentiation 4 cell; AIDS = Acquired immune deficiency syndrome; ART = Antiretroviral therapy.

**Table 2.** ^b^ Descriptive characteristics of the successful cognitive aging, vascular, and depression variables in the study sample of 136 PLWH.

	V−D−	V+D−	V+D+
*N* = 136	32	74	30
Vascular Conditions	0.1 (0.3)	3.9 (2.0)	4.2 (1.8)
POMS Depression/Dejection	4.7 (5.5)	7.2 (6.6)	25.7 (13.5)
Major Depressive Disorder (lifetime)	59.4%	63.5%	90.0%
Neurocognitive Global Deficit Score	0.2 (0.3)	0.4 (0.5)	0.4 (0.5)
Cognitive Symptoms			
POMS Confusion/Bewilderment	5.9 (3.4)	7.5 (4.2)	15.3 (5.3)
PRMQ	31.6 (8.4)	35.6 (11.2)	40.2 (11.6)
FrSBe Disinhibition	26.4 (6.7)	26.9 (6.7)	28.5 (7.8)
FrSBe Executive Dysfunction	32.0 (9.0)	35.7 (8.4)	39.7 (8.0)

b Values are means (standard deviation) or valid sample % values. PLWH = people living with Human Immunodeficiency Virus; V = vascular; D = depression; FrSBe = Frontal Systems Behavioral Scale POMS = Profile of Mood States; PRMQ = The Prospective and Retrospective Memory Questionnaire.

## References

[1] Aguero-Torres H, von Strauss E, Viitanen M, Winblad B, Fratiglioni L. Institutionalization in the elderly: The role of chronic diseases and dementia. Cross-sectional and longitudinal data from a population-based study. J Clin Epidemiol. 2001;54(8):795–801. doi: 10.1016/s0895-4356(00)00371-1 11470388

[2] Aizenstein HJ, Baskys A, Boldrini M, Butters MA, Diniz BS, Jaiswal MK, Jellinger KA, Kruglov LS, Meshandin IA, Mijajlovic MD, Niklewski G, Pospos S, Raju K, Richter K, Steffens DC, Taylor WD, Tene O. Vascular depression consensus report - a critical update. BMC Med. 2016;14(1):161. doi: 10.1186/s12916-016-0720-5 27806704 PMC5093970

[3] Alexopoulos GS, Meyers BS, Young RC, Campbell S, Silbersweig D, Charlson M. ‘Vascular depression’ hypothesis. Arch Gen Psychiatry. 1997;54(10):915–922. doi: 10.1001/archpsyc.1997.01830220033006 9337771

[4] Armitage P. Tests for linear trends in proportions and frequencies. Biometrics. 1955;11:375–386. doi: 10.2307/3001775

[5] Babu H, Ambikan AT, Gabriel EE, Svensson Akusjarvi S, Palaniappan AN, Sundaraj V, Mupanni NR, Sperk M, Cheedarla N, Sridhar R, Tripathy SP, Nowak P, Hanna LE, Neogi U. Systemic inflammation and the increased risk of inflamm-aging and age-associated diseases in people living with HIV on long-term suppressive antiretroviral therapy. Front Immunol. 2019;10:1965. doi: 10.3389/fimmu.2019.01965 31507593 PMC6718454

[6] Basavaraj KH, Navya MA, Rashmi R. Quality of life in HIV/AIDS. Indian J Sex Transm Dis AIDS. 2010;31(2):75–80. doi: 10.4103/2589-0557.74971 21716787 PMC3122586

[7] Beltran-Najera I, Mustafa A, Warren D, Salling Z, Misiura M, Woods SP, Dotson VM. Elevated frequency and everyday functioning implications of vascular depression in persons with HIV disease. J Psychiatr Res. 2023;160:78–85. doi: 10.1016/j.jpsychires.2023.02.003 36780803 PMC10123762

[8] Bloch M. Frailty in people living with HIV. AIDS Res Ther. 2018;15(1):19. doi: 10.1186/s12981-018-0210-2 30445966 PMC6240180

[9] Bogoian HR, Dotson VM. Vascular depression in Black Americans: A systematic review of the construct and its cognitive, functional, and psychosocial correlates. Clin Neuropsychol. 2022;36(2):431–461. doi: 10.1080/13854046.2021.1933188 34098846 PMC10450356

[10] Bonelli RM, Cummings JL. Frontal-subcortical circuitry and behavior. Dialogues Clin Neurosci. 2007;9(2):141–151. doi: 10.31887/DCNS.2007.9.2/rbonelli 17726913 PMC3181854

[11] Bryant VE, Whitehead NE, Burrell LE, Dotson VM, Cook RL, Malloy P, Devlin K, Cohen RA. Depression and apathy among people living with HIV: Implications for treatment of HIV-associated neurocognitive disorders. AIDS Behav. 2015;19(8):1430–1437. doi: 10.1007/s10461-014-0970-1 25533921 PMC4508233

[12] Casaletto KB, Umlauf A, Beaumont J, Gershon R, Slotkin J, Akshoomoff N, Heaton RK. Demographically corrected normative standards for the English version of the NIH Toolbox Cognition Battery. J Int Neuropsychol Soc. 2015;21:378–391. doi: 10.1017/S1355617715000351 26030001 PMC4490030

[13] Castilho JL, Rebeiro PF, Shepherd BE, Nash R, Adams RS, Turner M, Furukawa SS, Hulgan T, Koethe JR, Sterling TR. Mood disorders and increased risk of non-communicable disease in adults with HIV. J Acquir Immune Defic Syndr. 2021;83(4):397–404. doi: 10.1097/QAI.0000000000002269 PMC7594261 32097195

[14] Centers for Disease Control and Prevention (CDC). HIV in the United States by age. 2022. Retrieved from https://www.cdc.gov/hiv/group/age/index.html?CDC_AA_refVal=https%3A%2F%2Fwww.cdc.gov%2Fhiv%2Fgroup%2Fage%2Folderamericans%2Findex.html

[15] Chichetto NE, Kundu S, Freiberg MS, Koethe JR, Butt AA, Crystal S, So-Armah KA, Cook RL, Braithwaite RS, Justice AC, Fiellin DA, Khan M, Bryant KJ, Gaither JR, Barve SS, Crothers K, Bedimo RJ, Warner A, Tindle HA, Veterans Aging Cohort Study. Association of syndemic unhealthy alcohol use, smoking, and depressive symptoms on incident cardiovascular disease among veterans with and without HIV-infection. AIDS Behav. 2021;25(9):2852–2862. doi: 10.1007/s10461-021-03327-4 34101074 PMC8376776

[16] Cysique LA, Brew BJ. Vascular cognitive impairment and HIV-associated neurocognitive disorder: A new paradigm. J Neurovirol. 2019;25(5):710–721. doi: 10.1007/s13365-018-0706-5 30635846

[17] Deeks SG, Tracy R, Douek DC. Systemic effects of inflammation on health during chronic HIV infection. Immunity. 2013;39(4):633–645. doi: 10.1016/j.immuni.2013.10.001 24138880 PMC4012895

[18] Deng L, Zhang X, Gao Y, Turner D, Qian F, Lu H, Vermund SH, Zhang Y, Qian HZ. Association of HIV infection and cognitive impairment in older adults: A meta-analysis. Ageing Res Rev. 2021;68:101310. doi: 10.1016/j.arr.2021.101310 33640473 PMC10767715

[19] Derry HM, Johnston CD, Burchett CO, Brennan-Ing M, Karpiak S, Zhu YS, Siegler EL, Glesby MJ. Links between inflammation, mood, and physical function among older adults with HIV. J Gerontol B Psychol Sci Soc Sci. 2022;77(1):50–60. doi: 10.1093/geronb/gbab027 33580236 PMC8755907

[20] DeVaughn S, Müller-Oehring EM, Markey B, Bronte-Stewart HM, Schulte T. Aging with HIV-1 infection: Motor functions, cognition, and attention--a comparison with Parkinson’s disease. Neuropsychol Rev. 2015;25(4):424–438. doi: 10.1007/s11065-015-9305-x 26577508 PMC5519342

[21] Dew MA, Becker JT, Sanchez J, Caldararo R, Lopez OL, Wess J, Dorst SK, Banks G. Prevalence and predictors of depressive, anxiety and substance use disorders in HIV-infected and uninfected men: A longitudinal evaluation. Psychol Med. 1997;27(2):395–409. doi: 10.1017/s0033291796004552 9089832

[22] Dotson VM, Zonderman AB, Kraut MA, Resnick SM. Temporal relationships between depressive symptoms and white matter hyperintensities in older men and women. Int J Geriatr Psychiatry. 2013;28(1):66–74. doi: 10.1002/gps.3791 22415749 PMC3851322

[23] Duprez DA, Neuhaus J, Kuller LH, Tracy R, Belloso W, De Wit S, Drummond F, Lane HC, Ledergerber B, Lundgren J, Nixon D, Paton NI, Prineas RJ, Neaton JD, Group ISS. Inflammation, coagulation and cardiovascular disease in HIV-infected individuals. PLoS One. 2012;7(9):e44454. doi: 10.1371/journal.pone.0044454 22970224 PMC3438173

[24] Erlandson KM, Wilson MP, MaWhinney S, Rapaport E, Liu J, Wilson CC, Rahkola JT, Janoff EN, Brown TT, Campbell TB, Jankowski CM. The impact of moderate or high-intensity combined exercise on systemic inflammation among older persons with and without HIV. J Infect Dis. 2021;223(7):1161–1170. doi: 10.1093/infdis/jiaa494 32779711 PMC8030723

[25] Fazeli PL, Woods SP, Vance DE. Successful functional aging in middle-aged and older adults with HIV. AIDS Behav. 2020;24(6):1592–1598. doi: 10.1007/s10461-019-02635-0 31414298 PMC7018561

[26] Fazeli PL, Willig AL, Oliveira V, Buford TW, Vance DE, Burkholder G, Crane HM, Horvat Davey C, Fleming J, Webel AR. The association between objectively-measured physical activity and cognitive functioning in middle-aged and older people living with HIV. AIDS Behav. 2023;27(4):1199–1210. doi: 10.1007/s10461-022-03857-5 36163604 PMC10129017

[27] Ferrucci L, Fabbri E. Inflammageing: Chronic inflammation in ageing, cardiovascular disease, and frailty. Nat Rev Cardiol. 2018;15(9):505–522. doi: 10.1038/s41569-018-0064-2 30065258 PMC6146930

[28] Field-Fote E. Mediators and moderators, confounders, and covariates: Exploring the variables that illuminate or obscure the “active ingredients” in neurorehabilitation. J Neurol Phys Ther. 2019;43(2):83–84. doi: 10.1097/NPT.0000000000000275 30883494

[29] González HM, Tarraf W, Whitfield K, Gallo JJ. Vascular depression prevalence and epidemiology in the United States. J Psychiatr Res. 2012;46(4):456–461. doi: 10.1016/j.jpsychires.2012.01.011 22277303 PMC3447181

[30] Grace J, Malloy PF. Frontal Systems Behavior Scale. Professional manual. Lutz, FL: Psychological Assessment Resources, Inc.; 2001.

[31] Guaraldi G, Orlando G, Zona S, Menozzi M, Carli F, Garlassi E, Berti A, Rossi E, Roverato A, Palella F. Premature age-related comorbidities among HIV-infected persons compared with the general population. Clin Infect Dis. 2011;53(11):1120–1126. doi: 10.1093/cid/cir627 21998278

[32] Guha A, Brier MR, Ortega M, Westerhaus E, Nelson B, Ances BM. Topographies of cortical and subcortical volume loss in HIV and aging in the cART era. J Acquir Immune Defic Syndr. 2016;73(4):374–383. doi: 10.1097/QAI.0000000000001111 27454251 PMC5085858

[33] Ibeneme SC, Uwakwe VC, Myezwa H, Irem FO, Ezenwankwo FE, Ajidahun TA, Ezuma AD, Okonkwo UP, Fortwengel G. Impact of exercise training on symptoms of depression, physical activity level and social participation in people living with HIV/AIDS: A systematic review and meta-analysis. BMC Infect Dis. 2022;22(1):469. doi: 10.1186/s12879-022-07145-4 35578192 PMC9109396

[34] Iudicello JE, Woods SP, Deutsch R, Grant I, HIV Neurobehavioral Research Program Hnrp Group. Combined effects of aging and HIV infection on semantic verbal fluency: A view of the cortical hypothesis through the lens of clustering and switching. J Clin Exp Neuropsychol. 2012;34(5):476–488. doi: 10.1080/13803395.2011.651103 22292479 PMC3329578

[35] Jellinger KA. Pathomechanisms of vascular depression in older adults. Int J Mol Sci. 2021;23(1). doi: 10.3390/ijms23010308 35008732 PMC8745290

[36] Pro JMP, Version 16.0. SAS Institute Inc. Cary, NC, 1989-2021.

[37] Kirton JW, Resnick SM, Davatzikos C, Kraut MA, Dotson VM. Depressive symptoms, symptom dimensions, and white matter lesion volume in older adults: A longitudinal study. Am J Geriatr Psychiatry. 2014;22(12):1469–1477. doi: 10.1016/j.jagp.2013.10.005 24211028 PMC3984387

[38] Krishnan KR, Hays JC, Blazer DG. MRI-defined vascular depression. Am J Psychiatry. 1997;154(4):497–501. doi: 10.1176/ajp.154.4.497 9090336

[39] Kuhn T, Kaufmann T, Doan NT, Westlye LT, Jones J, Nunez RA, Bookheimer SY, Singer EJ, Hinkin CH, Thames AD. An augmented aging process in brain white matter in HIV. Hum Brain Mapp. 2018;39(6):2532–2540. doi: 10.1002/hbm.24019 29488278 PMC5951745

[40] Langebeek N, Kooij KW, Wit FW, Stolte IG, Sprangers MAG, Reiss P, Nieuwkerk PT, AGEhIV Cohort Study Group. Impact of comorbidity and ageing on health-related quality of life in HIV-positive and HIV-negative individuals. AIDS. 2017;31(10):1471–1481. doi: 10.1097/qad.0000000000001511 28574965

[41] Levy ME, Anastos K, Levine SR, Plankey M, Castel AD, Molock S, Sen S, Asch FM, Milam J, Aouizerat B, Weber KM, Golub ET, Kaplan RC, Kassaye S. Depression and psychosocial stress are associated with subclinical carotid atherosclerosis among women living with HIV. J Am Heart Assoc. 2020;9(13):e016425. doi: 10.1161/JAHA.120.016425 32564652 PMC7670495

[42] Malaspina L, Woods SP, Moore DJ, Depp C, Letendre SL, Jeste D, Grant I, The HIV Neurobehavioral Research Programs (HNRP) Group. Successful cognitive aging in persons living with HIV infection. J Neurovirol. 2011;17(1):110–119. doi: 10.1007/s13365-010-0008-z 21165783 PMC3032198

[43] Mayo NE, Brouillette M, Nadeau L, Dendukuri N, Harris M, Smaill F, Smith G, Thomas R, Fellows LK, Investigators from the Positive Brain Health Now Study. A longitudinal view of successful aging with HIV: Role of resilience and environmental factors. Qual Life Res. 2022;31(4):1135–1145. doi: 10.1007/s11136-021-02970-7 34460077

[44] McCutchan JA, Marquie-Beck JA, Fitzsimons CA, Letendre SL, Ellis RJ, Heaton RK, Wolfson T, Rosario D, Alexander TJ, Marra C, Ances BM, Grant I. Role of obesity, metabolic variables, and diabetes in HIV-associated neurocognitive disorder. Neurology. 2012;78(7):485–492. doi: 10.1212/WNL.0b013e3182478d64 22330412 PMC3280051

[45] McIntosh EC, Tureson K, Rotblatt LJ, Singer EJ, Thames AD. HIV, vascular risk factors, and cognition in the combination antiretroviral therapy era: A systematic review and meta-analysis. J Int Neuropsychol Soc. 2021;27(4):365–381. doi: 10.1017/S1355617720001022 33161930 PMC9618305

[46] McNair PM, Lorr M, Droppleman LF. POMS manual. 2nd ed. San Diego: Educational and Industrial Testing Service; 1981.

[47] Miller PE, Budoff M, Zikusoka M, Li X, Palella F Jr, Kingsley LA, Witt MD, Sharrett AR, Jacobson LP, Post WS. Comparison of racial differences in plaque composition and stenosis between HIV-positive and HIV-negative men from the Multicenter AIDS Cohort Study. Am J Cardiol. 2014;114(3):369–375. doi: 10.1016/j.amjcard.2014.04.049 24929623 PMC4143765

[48] Monteiro-Junior RS, de Tarso Maciel-Pinheiro P, da Matta Mello Portugal E, da Silva Figueiredo LF, Terra R, Carneiro LSF, Rodrigues VD, Nascimento OJM, Deslandes AC, Laks J. Effect of exercise on inflammatory profile of older persons: Systematic review and meta-analyses. J Phys Act Health. 2018;15(1):64–71. doi: 10.1123/jpah.2016-0735 28771081

[49] Moore RC, Fazeli PL, Jeste DV, Moore DJ, Grant I, Woods SP, The HIV Neurobehavioral Research Program (HNRP) Group. Successful cognitive aging and health-related quality of life in younger and older adults infected with HIV. AIDS Behav. 2014;18(6):1186–1197. doi: 10.1007/s10461-014-0743-x 24633788 PMC4020963

[50] Moore DJ, Fazeli PL, Moore RC, Woods SP, Letendre SL, Jeste DV, Grant I, The HIV Neurobehavioral Research Program (HNRP) Group. Positive psychological factors are linked to successful cognitive aging among older persons living with HIV/AIDS. AIDS Behav. 2018;22(5):1551–1561. doi: 10.1007/s10461-017-2001-5 29264737 PMC5903987

[51] Müller-Oehring EM, Hong JY, Poston KL, Brontë-Stewart HM, Sullivan EV, McGlynn L, Schulte T. Neurofunctional characteristics of executive control in older people with HIV infection: a comparison with Parkinson’s disease. Brain Imaging Behav. 2022;16(4):1776–1793. doi: 10.1007/s11682-022-00645-6 35294979 PMC10124990

[52] Neuhaus J, Jacobs DR Jr, Baker JV, Calmy A, Duprez D, La Rosa A, Kuller LH, Pett S, Ristola M, Ross MJ, Shlipak MG, Tracy R, Neaton JD. Markers of inflammation, coagulation, and renal function are elevated in adults with HIV infection. J Infect Dis. 2010;201(12):1788–1795. doi: 10.1086/652749 20446848 PMC2872049

[53] Nyenhuis DL, Yamamoto C, Luchetta T, Terrien A, Parmentier A. Adult and geriatric normative data and validation of the Profile of Mood States. J Clin Psychol. 1999;55(1):79–86. doi: 10.1002/(SICI)1097-4679(199901)55:1<79::AID-JCLP8>3.0.CO;2–7 10100834

[54] Oursler KK, Goulet JL, Crystal S, Justice AC, Crothers K, Butt AA, Rodriguez-Barradas MC, Favors K, Leaf D, Katzel LI, Sorkin JD. Association of age and comorbidity with physical function in HIV-infected and uninfected patients: Results from the Veterans Aging Cohort Study. AIDS Patient Care STDS. 2011;25(1):13–20. doi: 10.1089/apc.2010.0242 21214375 PMC3030913

[55] Parruti G, Vadini F, Sozio F, Mazzott E, Ursini T, Polill E, Di Stefano P, Tontodonati M, Verrocchio MC, Fulcheri M, Calella G, Santilli F, Manzoli L. Psychological factors, including alexithymia, in the prediction of cardiovascular risk in HIV-infected patients: Results of a cohort study. PLoS One. 2013;8(1):e54555. doi: 10.1371/journal.pone.0054555 23349927 PMC3551818

[56] Patterson K, Young C, Woods SP, Vigil O, Grant I, Atkinson JH, The HIV Neurobehavioral Research Center (HNRC) Group. Screening for major depression in persons with HIV infection: The concurrent predictive validity of the Profile of Mood States Depression-Dejection Scale. Int J Methods Psychiatr Res. 2006;15(2):75–82. doi: 10.1002/mpr.184 19722288 PMC6878440

[57] Petersen KJ, Metcalf N, Cooley S, Tomov D, Vaida F, Paul R, Ances BM. Accelerated brain aging and cerebral blood flow reduction in persons with Human Immunodeficiency Virus. Clin Infect Dis. 2021;73(10):1813–1821. doi: 10.1093/cid/ciab169 33621317 PMC8599198

[58] Qin P, He J, Yang X, Chen S, Chen X, Jiang H, Fung AWT, Wang Z, Lau JTF. The role of depressive symptoms and physical activity levels in mediating the association between HIV status and neurocognitive functions among individuals aged at least 50 years in China: Cross-sectional study. JMIR Public Health Surveill. 2022;8(8):e32968. doi: 10.2196/32968 35984684 PMC9440416

[59] Rabkin JG. HIV and depression: 2008 review and update. Curr HIV/AIDS Rep. 2008;5(4):163–171. doi: 10.1007/s11904-008-0025-1 18838056

[60] Rodriguez-Penney AT, Ludicello JE, Riggs PK, Doyle K, Ellis RJ, Letendre SL, Grant I, Woods SP; HIV Neurobehavioral Research Program (HNRP) Group. Co-morbidities in persons infected with HIV: Increased burden with older age and negative effects on health-related quality of life. AIDS Patient Care STDS. 2013 Jan;27(1):5–16. doi: 10.1089/apc.2012.0329. 23305257 PMC3545369

[61] Roman GC, Jackson RE, Gadhia R, Roman AN, Reis J. Mediterranean diet: The role of long-chain omega-3 fatty acids in fish; polyphenols in fruits, vegetables, cereals, coffee, tea, cacao and wine; probiotics and vitamins in prevention of stroke, age-related cognitive decline, and Alzheimer disease. Rev Neurol (Paris). 2019 Dec;175(10):724–741. doi: 10.1016/j.neurol.2019.08.005. 31521398

[62] Rooney AS, Moore RC, Paolillo EW, Gouaux B, Umlauf A, Letendre SL, Jeste DV, Moore DJ, Program HINVNRP. Depression and aging with HIV: Associations with health-related quality of life and positive psychological factors. J Affect Disord. 2019 Jul 15;251:1–7. doi: 10.1016/j.jad.2019.03.025. 30884371 PMC6705595

[63] Rubin LH, Benning L, Keating SM, Norris PJ, Burke-Miller J, Savarese A, Kumanan KN, Awadalla S, Springer G, Anastos K, Young M, Milam J, Valcour VG, Weber KM, Maki PM. Variability in C-reactive protein is associated with cognitive impairment in women living with and without HIV: a longitudinal study. J Neurovirol. 2018 Feb;24(1):41–51. doi: 10.1007/s13365-017-0590-4. 29063513 PMC6036635

[64] Rubtsova AA, Wingood GM, Ofotokun I, Gustafson D, Vance DE, Sharma A, Adimora AA, Holstad M. Prevalence and correlates of self-rated successful aging among older women living with HIV. J Acquir Immune Defic Syndr. 2019 Jun 1;82(2):S162–S169. doi: 10.1097/QAI.0000000000002175. PMC6830959 31658205

[65] Saloner R, Campbell LM, Serrano V, Montoya JL, Pasipanodya E, Paolillo EW, Franklin D, Ellis RJ, Letendre SL, Collier AC, Clifford DB, Gelman BB, Marra CM, McCutchan JA, Morgello S, Sacktor N, Jeste DV, Grant I, Heaton RK, Moore DJ, ; CHARTER and HNRP Groups. Neurocognitive SuperAging in older adults living with HIV: Demographic, neuromedical and everyday functioning correlates. J Int Neuropsychol Soc. 2019 May;25(5):507–519. doi: 10.1017/S1355617719000018. PMC6705613 30890191

[66] Sheppard DP, Woods SP, Bondi MW, Gilbert PE, Massman PJ, Doyle KL; HIV Neurobehavioral Research Program Group. Does older age confer an increased risk of incident neurocognitive disorders among persons living with HIV disease? Clin Neuropsychol. 2015 Jul;29(5):656–677. doi: 10.1080/13854046.2015.1077995. PMC4570033 26367342

[67] Sheppard DP, Woods SP, Massman PJ, Gilbert PE. Frequency and correlates of subjective cognitive impairment in HIV disease. AIDS Behav. 2019 Mar;23(3):617–626. doi: 10.1007/s10461-018-2297-9. 30311103 PMC6481638

[68] Shimizu SM, Chow DC, Valcour V, Masaki K, Nakamoto BK, Kallianpur KJ, Shikuma C. The impact of depressive symptoms of neuropsychological performance tests in HIV-infected individuals: A study of the Hawaii aging with HIV cohort. World J AIDS. 2011 Dec;1(4):139–145. doi: 10.4236/wja.2011.14020. 23061029 PMC3467015

[69] Sico JJ, Kundu S, So-Armah K, Gupta SK, Chang CH, Butt AA, Gilbert CL, Marconi VC, Crystal S, Tindle HA, Freiberg MS, Stewart JC. Depression as a risk factor for incident ischemic stroke among HIV-positive veterans in the Veterans Aging Cohort Study. J Am Heart Assoc. 2021 Jul;10(13):e017637. doi: 10.1161/JAHA.119.017637. 34169726 PMC8403311

[70] Siedner MJ, Zanni M, Tracy RP, Kwon DS, Tsai AC, Kakuhire B, Hunt PW, Okello S. Increased systemic inflammation and gut permeability among women with treated HIV infection in rural Uganda. J Infect Dis. 2018 Sep;218(6):922–926. doi: 10.1093/infdis/jiy244. 29718342 PMC6093434

[71] Smit M, Brinkman K, Geerlings S, Smit C, Thyagarajan K, Sighem A, de Wolf F, Hallett TB, ATHENA Observational Cohort. Future challenges for clinical care of an ageing population infected with HIV: A modelling study. Lancet Infect Dis. 2015 Aug;15:810–818. doi: 10.1016/S1473-3099(15)00056-0. 26070969 PMC4528076

[72] Smith G, Della Sala S, Logie RH, Maylor EA. Prospective and retrospective memory in normal aging and dementia: A questionnaire study. Memory. 2000 Sep-Oct;8(5):311–321. doi: 10.1080/09658210050117735. 11045239

[73] So-Armah K, Benjamin LA, Bloomfield GS, Feinstein MJ, Hsue P, Njuguna B, Freiberg MS. HIV and cardiovascular disease. Lancet HIV. 2020 Apr;7(4):e279–e293. doi: 10.1016/S2352-3018(20)30036-9. 32243826 PMC9346572

[74] Stradling C, Thomas GN, Hemming K, Taylor S, Taheri S. Randomized parallel-group pilot trial (Best foods for your heart) comparing the effects of a Mediterranean Portfolio diet with a low saturated fat diet on HIV dyslipidemia. Clin Nutr. 2021 Mar;40(3):860–869. doi: 10.1016/j.clnu.2020.08.038. 33032837

[75] Thompson JL, Sheppard DP, Matchanova A, Morgan EE, Loft S, Woods SP. Subjective cognitive decline disrupts aspects of prospective memory in older adults with HIV disease. Neuropsychol Dev Cogn B Aging Neuropsychol Cogn. 2022 Mar;29(2):308–326. doi: 10.1080/13825585.2022.2065241. PMC9554043 35412440

[76] Tierney SM, Woods SP, Sheppard D, Ellis RJ. Extrapyramidal motor signs in older adults with HIV disease: Frequency, 1-year course, and associations with activities of daily living and quality of life. J Neurovirol. 2019 Apr;25(2):162–173. doi: 10.1007/s13365-018-0699-0. 30535869

[77] Tomaszewski Farias S, Cahn-Weiner DA, Harvey DJ, Reed BR, Mungas D, Kramer JH, Chui H. Longitudinal changes in memory and executive functioning are associated with longitudinal change in instrumental activities of daily living in older adults. Clin Neuropsychol. 2009 Apr;23(3):446–461. doi: 10.1080/13854040802360558. 18821181 PMC2881703

[78] Umegaki H, Sakurai T, Arai H. Active life for brain health: A narrative review of the mechanism underlying the protective effects of physical activity on the brain. Front Aging Neurosci. 2021;13:761674. doi: 10.3389/fnagi.2021.761674. 34916925 PMC8670095

[79] Valcour V, Shikuma C, Shiramizu B, Poff P, Selnes O, Holck P, Grove J, Sacktor N. Higher frequency of dementia in older HIV-1 individuals: The Hawaii aging with HIV-1 cohort. Neurology. 2004 Sep 14;63(5):822–827. doi: 10.1212/01.wnl.0000134665.58343.8d. 15365130 PMC1382180

[80] Vance DE, Blake BJ, Brennan-Ing M, DeMarco RF, Fazeli PL, Relf MV. Revisiting successful aging with HIV through a revised biopsychosocial model: An update of the literature. J Assoc Nurses AIDS Care. 2019 Jan-Feb;30(1):5–14. doi: 10.1097/jnc.0000000000000029. 30586079

[81] Vance DE, Lee Y, Batey DS, Li W, Chapman Lambert C, Nakkina SR, Anderson JN, Triebel K, Byun JY, Fazeli PL. Emerging directions of cognitive aging with HIV: Practice and policy implications for social work. J Gerontol Soc Work. 2022;65(5):476–494. doi: 10.1080/01634372.2021.1978028. 34511048

[82] Wallace LMK, Ferrara M, Brothers TD, Garlassi S, Kirkland SA, Theou O, Zona S, Mussini C, Moore D, Rockwood K, Guaraldi G. Physical frailty and risk of cognitive impairment in HIV-infected individuals. AIDS Res Hum Retroviruses. 2017 Feb;33(2):157–163. doi: 10.1089/aid.2016.0189. 27869500 PMC5335777

[83] Woods SP, Dawson MS, Weber E, Grant I; HIV Neurobehavioral Research Center (HNRC) Group. The semantic relatedness of cue-intention pairings influences event-based prospective memory failures in older adults with HIV infection. J Clin Exp Neuropsychol. 2010 Apr;32(4):398–407. doi: 10.1080/13803390903130737. 19763997 PMC2854853

[84] Woods SP, Morgan EE, Loft S, Matchanova A, Verduzco M, Cushman C. Supporting strategic processes can improve time-based prospective memory in the laboratory among older adults with HIV disease. Neuropsychology. 2020 Apr;34(3):249–263. doi: 10.1037/neu0000602. PMC7042030 31789564

[85] World Health Organization (WHO). Composite international diagnostic interview (CIDI, version 2.128). Geneva, Switzerland: World Health Organization; 1998.

